# Characterizing Human Stem Cell–derived Sensory Neurons at the Single-cell Level Reveals Their Ion Channel Expression and Utility in Pain Research

**DOI:** 10.1038/mt.2014.86

**Published:** 2014-06-17

**Authors:** Gareth T Young, Alex Gutteridge, Heather DE Fox, Anna L Wilbrey, Lishuang Cao, Lily T Cho, Adam R Brown, Caroline L Benn, Laura R Kammonen, Julia H Friedman, Magda Bictash, Paul Whiting, James G Bilsland, Edward B Stevens

**Affiliations:** 1 Pfizer Neusentis, Cambridge, UK; 2 Current address: Department of Physiology, Anatomy and Genetics, University of Oxford, Oxford, UK; 3 Oncology Research Unit, Pfizer Global Research and Development, Pearl River, NY, USA

## Abstract

The generation of human sensory neurons by directed differentiation of pluripotent stem cells opens new opportunities for investigating the biology of pain. The inability to generate this cell type has meant that up until now their study has been reliant on the use of rodent models. Here, we use a combination of population and single-cell techniques to perform a detailed molecular, electrophysiological, and pharmacological phenotyping of sensory neurons derived from human embryonic stem cells. We describe the evolution of cell populations over 6 weeks of directed differentiation; a process that results in the generation of a largely homogeneous population of neurons that are both molecularly and functionally comparable to human sensory neurons derived from mature dorsal root ganglia. This work opens the prospect of using pluripotent stem-cell–derived sensory neurons to study human neuronal physiology and as *in vitro* models for drug discovery in pain and sensory disorders.

## Introduction

The *in vitro* generation of differentiated cells from pluripotent stem cells is a key goal of regenerative medicine. Such cells can be used in place of human tissue or animal models for disease modeling, drug screening, and even cell replacement therapies.^1^ A major challenge, addressed here, is improving the level of molecular and cellular understanding of the *in vitro* differentiation process and in depth functional characterization of the terminally differentiated cells produced.

Much research has focused on differentiating pluripotent cells into neurons of the brain^2,3,4^ and those neuronal subtypes central to neurodegenerative diseases such as Parkinson's disease^5,6,7^ and amyotrophic lateral sclerosis.^8^ A relative scarcity of protocols exist that describe the derivation of sensory neurons of the peripheral nervous system.^9,10^ The lack of access to this tissue has limited our understanding of its development and the physiology of pain in humans.

We recently reported a small molecule differentiation protocol, which results in the differentiation of human pluripotent stem cells (hPSC) to sensory neurons of a nociceptor phenotype^9^ (here named hPSC-sensory). This protocol involves dual SMAD inhibition (2i-LDN193189, SB43152), which we have previously shown efficiently induces neuroectoderm formation from human embryonic stem cells.^11^ This is followed by inhibition of GSK-3, γ-secretase, and vascular endothelial growth factor receptor/fibroblast growth factor receptor (3i-CHIR99021, DAPT, SU5402), which enables fate specification toward a sensory phenotype followed by maturation of the neurons with growth factors (brain-derived neurotrophic factor, glial derived neurotrophic factor, neuronal growth factor, and NT3 supplemented with ascorbic acid). This protocol generates cells expressing canonical markers of sensory neurons such as *TAC1* (substance P/neurokinin A precursor), *SLC17A6* (VGLUT2), *SCN9A* (Na_V_1.7), and *SCN10A* (Na_V_1.8). Electrophysiological recordings also revealed the expression of the nociceptor expressed ion channels Na_v_1.8 and P2X3 (ref. 9).

To build on this work, we have performed an in-depth characterization of the gene expression changes accompanying the differentiation of hPSC-sensory. Importantly, for the first time, we compare the time course of the global gene expression profile to that of the relevant primary human tissue, human dorsal root ganglia (hDRG), and found the profile of hPSC-sensory to be highly comparable to hDRG by 30 days differentiation *in vitro*. To complement this population level data, we also performed single-cell quantitative polymerase chain reaction (qPCR) at five time points during differentiation and used these data to track the development and decline of intermediate cell types and to assess the homogeneity of cells within the culture.

Although molecular and cellular assays are important for establishing the success of a differentiation protocol, the suitability of the cellular product for disease modeling is ultimately decided by its functionality. For neurons, function is determined by the complement of ion channels they express and the nature of the action potentials they generate. We found that the ion channel expression profile in hPSC-sensory was close to that of adult hDRG (84% of hDRG ion channel genes were expressed in hPSC-sensory), so we then characterized hPSC-sensory using patch-clamp electrophysiology and pharmacological modulation. We found functional expression of key ion channels associated with pain and sensory disorders such as ASIC1/2, HCN1, GABA_A_R, KCNQ2/3, and TRPV1. Pharmacological molecules were employed in this study to assess which ion channel subunits are expressed in hPSC-sensory; these molecules have not previously been assessed in human neurons thus providing novel information on their activity.

The principle of using molecular analysis coupled with functional characterization of differentiated cells is one with broad application in regenerative medicine. In this specific case, our data provide an in-depth characterization of hPSC-sensory, demonstrating for the first time, the similarities between cells derived *in vitro* from our protocol and hDRG. This work opens new avenues for research into pain and sensory disease modeling, target validation, and *in vitro* screening for the next generation of analgesic drugs.

## Results

### Generation of hPSC-sensory from small molecule inhibition of BMP, GSK3, γ-secretase, vascular endothelial growth factor receptor, and fibroblast growth factor receptor followed by growth in neurotrophin media

**Figure 1:**
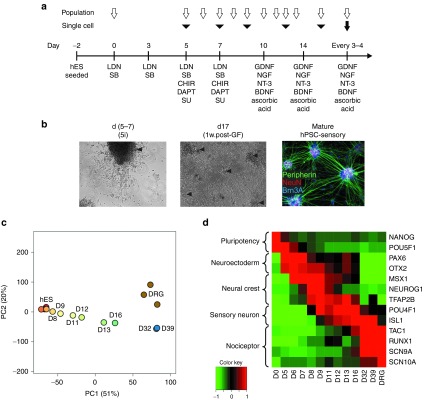
**Directed differentiation produces neurons with a nociceptor phenotype**. (**a**) Directed differentiation protocol for human pluripotent stem cells (hPSC)-sensory. LDN193189 (LDN), SB-431542 (SB), CHIR99021 (CHIR), and SU5402 (SU). Open arrows denote timing of microarray analysis, black arrowheads denote timing of single cell qPCR analysis, and the black arrow denotes timing of electrophysiological analysis. (**b**) Phase contrast images of hPSC-sensory through the differentiation protocol. Cellular morphology after 5i is seen in left panel with emerging islands of neuronal cell bodies (arrow head). After growth factor addition many neuronal islands can be seen (arrow heads) which stain positive for sensory neuronal markers peripherin, NeuN, and Brn3a (right panel), scale bar 400 µm. (**c**) Sample clustering based on a principal components analysis of genome-wide expression levels across all samples. Although measured in triplicate, for clarity each time point is represented by a single sample comprising the median expression level. Samples from the differentiating hES are colored red through blue according to the time of sampling. hDRG samples are colored brown. Key time points are labeled. The percentage of total variation accounted for by each principal component is given in parentheses. (**d**) Relative expression levels of key marker genes across the time course on log_2_ scale. The median expression levels of each gene across replicates are mean centered and scaled to unit variance. BDNF, brain-derived neurotrophic factor; GDNF, glial derived neurotrophic factor; hES, human embryonic stem cell; NGF, neuronal growth factor.

We have previously reported generation of functional sensory-like neurons from human embryonic stem cell (hES) cells using a cocktail of small molecule developmental pathway inhibitors^9^; a slightly modified protocol was used here (see **Figure 1a** and Materials and Methods). Under these conditions, the cells expanded during the SMAD inhibition phase, followed by differentiation into colonies of neural precursors and death of nonneural cells during the five inhibitor phase (**Figure 1b**). Neural colonies exhibited neurite outgrowth (**Figure 1b**) and labeled with the sensory markers peripherin, Islet-1, and Brn3A, confirming their sensory identity. We compared the differentiation protocol used here to that previously reported^9^ by assaying hPSC-sensory by qPCR, immunocytochemical, and electrophysiological assessments. The protocol used here to generate hPSC-sensory express nociceptor specific genes with a temporal profile comparable to that previously observed (**Supplementary Figure S2**). Both protocols generated neurons which stain for peripherin, Islet-1, and Brn3A 1 week following growth factor addition (**Supplementary Table S3**). Both protocols generate hPSC-sensory which respond to capsaicin (TRPV1 +ve) after 6 weeks in growth factor containing media (**Supplementary Table S3**). Finally, both differentiation protocols generate hPSC-sensory with comparable voltage-gated sodium currents after 2 weeks in growth factor–containing media (**Supplementary Table S3** and **Supplementary Figure S2**), which could fire multiple action potentials (16 out of 52 cells tested) and were blocked by a Nav1.8-selective blocker A-803467 (control: 5.8 ± 0.1 Hz, A-803467-treated: 1.0 ± 0.0 Hz; *n* = 4), reflecting Na_v_1.8 expression.

### hPSC-sensory express sensory neuronal genes and have comparable expression to human DRG

The production of differentiated cells for *in vitro* disease modeling requires robust, reproducible differentiation protocols that produce cells comparable to the clinically relevant *in vivo* cell type as an end product. To better understand the identity of the cells being produced in our protocol, we characterized the transcriptome of hPSC-sensory with whole-genome microarrays at 12 time points during their differentiation from hES to hPSC-sensory. In order to compare hPSC-sensory with the most relevant primary human tissue, we also profiled DRG taken from three separate adult donors.

Principal components analysis^12,13^ was used to visualize the similarities between hPSC-sensory and hDRG. A plot of the first two principal components is shown in **Figure 1c**. The clustering of the samples clearly demonstrates the increasing similarity between hPSC-sensory and the hDRG population over time. It is also notable that, while there is a large difference in global gene expression between cells sampled at day 16 and day 32, there is very little difference between day 32 and day 39. A differential expression analysis indicates that only 73 genes undergo a change in expression greater than twofold between day 32 and day 39 (compared to 2,347 between day 16 and day 32), suggesting that maturation at the level of gene transcription has largely plateaued by these later time points.

A gene set enrichment analysis^14,15,16^ of genes differentially expressed between the most mature hPSC-sensory samples and hDRG shows that the differences are mostly accounted for by expression of genes involved in the formation of the fatty myelin sheath and inflammatory processes (see **Supplementary Table S1** for a full list of genes enriched in the hDRG). These genes are likely to originate from myelin-producing Schwann cells and infiltrating immune cells found in the hDRG samples. An advantage of using *in vitro*-derived neurons over primary tissue in this case is the higher purity of the cells and the avoidance of potential confounding factors introduced by the interactions of non-neuronal cells with the neurons.

Next, we surveyed the expression of key pluripotency, neuroectoderm, neural crest, sensory neuronal, and nociceptive markers during the differentiation time course (**Figure 1d**). The expression levels of these markers in hDRG are shown for comparison. The pluripotency markers (*NANOG* and *POU5F1*)^17^ drop in expression up to day 2, while neuroectoderm markers such as *PAX6* and neural crest markers such as *MSX1* and *NEUROG1* peak and then fall away between days 6 and 9. This is followed by steadily increasing expression of more specific neuronal markers such as *POU4F1* (Brn3a). Expression of sensory neuron-specific markers, such as the ion channel *SCN9A* (Na_V_1.7), the transcription factor RUNX1 and the peptide precursor *TAC1*, in the later stage of differentiation indicates the development of this specific neuronal type. We note that statistically significant levels of *SCN10A* (Na_V_1.8) were not detected by the microarray, but a low level of expression was observed by a more sensitive qPCR-based expression assay (**Figure 4c**) and previously by patch-clamp electrophysiology.^9^

### Single-cell analysis reveals the degree of heterogeneity in terminally differentiated hPSC-sensory

**Figure 2:**
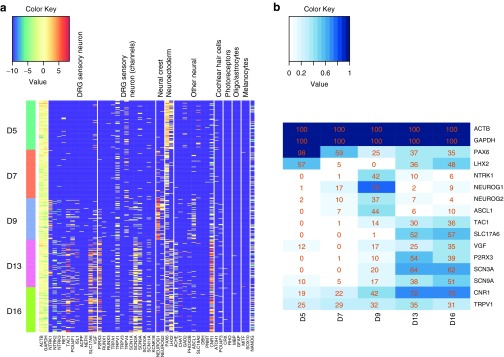
**Expression of key marker genes at single cell resolution measured by qPCR**. (**a**) Each row represents the expression levels measured from a single cell taken from the given time point. The measured genes are categorized according to the functional groupings given. In the case of no detectable expression, a color corresponding to the lowest level of detection is given (purple). (**b**) The percentage of cells with any detectable expression of the given genes at each time point. Genes were selected on the basis that expression was detected in >33% of cells at any one time point.

While microarray data provide a detailed, population-wide readout of developmental state, a crucial piece of information not provided is a measure of cellular heterogeneity within the culture. To address this, we performed single-cell qPCR at five time points in the differentiation using 48 genes including housekeepers, key neuronal fate markers, and markers of alternative fates. In total, 396 cells were profiled across the time points. **Figure 2a** shows the totality of the expression data. Clear differences in gene expression between the early (days 5 and 7), intermediate (day 9), and late (day 13 and 16) time points are visible, **Figure 2b** summarizes these changes in terms of a subset of key markers.

We find that the early population of single cells is mostly positive for neuroectoderm markers *PAX6* and *LHX2* (98 and 57%, respectively). The proportion of *PAX6*^+^ and *LHX2*^+^ cells drops significantly at mid time points (no *LHX2*^*+*^ cells were found at day 9) after which it increases slightly again. These changes are recapitulated in the microarray data (see **Supplementary Figure S1a**), but the single-cell data allow us to show that *PAX6* expression is not in fact present in neuronal cells at these later time points. Rather, it is a marker of a non-neuronal cell population. Another advantage of the single cell analysis is the ability to see precisely which genes are coexpressed in individual cells. Surprisingly, there is no detectable correlation at the late time points between *PAX6* and *LHX2* expression levels at the level of individual cells. This indicates that the residual *PAX6*^+^ and *LHX2*^+^ populations observed at these later time points are themselves distinct.

The neural crest markers *NEUROG1*, *NEUROG2*, and *NTRK1* are evident at day 9 where 75% of cells are *NEUROG1*^+^ (compared to 17% at day 7 and just 2% at day 13). The sharp rise and fall in expression is recapitulated in the microarray data (**Supplementary Figure S1b**). Unlike *PAX6* and *LHX2*, the expression of these neural crest markers is weakly correlated at the individual cell level (*R* = 0.29; *P* < 0.05) at day 9. Expression of *NEUROG1* is however higher than *NEUROG2* in absolute terms in almost all cells and the proportion of *NEUROG1*^+^ cells (75%) is considerably higher than *NEUROG2*^+^ (37%). This pattern of expression would be consistent with the formation of sensory neurons from the unmyelinated lineage.^18^

At the later time points, the majority of cells express neuronal markers such as *CNR1*, but also sensory neuron specific genes such as *SLC17A6* and *SCN9A*. Expression of these genes tends to correlate at the single-cell level, pointing toward a relatively homogenous population. Consistent with the microarray data, we do not observe significant numbers of cells expressing *RUNX1* or *SCN10A* by day 16 indicating that these markers require additional maturation time. Almost no expression of markers for other terminally differentiated cell types such as cochlear hair cells, photoreceptors, oligodendrocytes, astrocytes, or melanocytes was observed.

**Figure 3:**
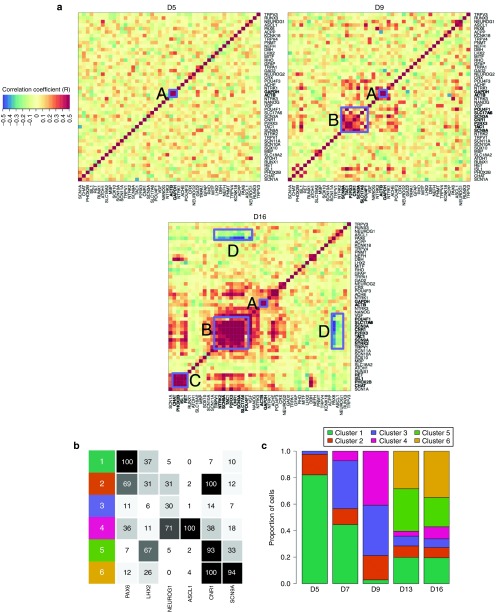
**Intergene expression correlations and single-cell clustering**. (**a**) Genes are clustered by hierarchical single linkage clustering based on the Euclidean distance between rows of the day 16 matrix. Red squares indicate high intracellular correlation between expression levels of pairs of genes. Four groupings of genes mentioned in the text are highlighted: (A) housekeeping genes; (B) sensory neuron markers; (C) sympathetic markers; (D) neuroectodermal and neural crest markers. (**b**) The percentage of cells in each of the six clusters of cells (1–6) identified by PAM expressing the given marker gene. (**c**) The proportion of all cells at each timepoint included in each cluster of cells.

**Figure 3a** shows a heatmap representing the pairwise gene correlations observed in cells sampled at days 5, 9, and 16. The most obvious effect is the emergence of intergene correlations as the differentiation progresses. Very little correlation between genes is visible at day 5 compared to day 16. This simply reflects the fact that the genes chosen for the assay are primarily mature neuronal markers, and so, most genes are simply not detected at day 5 (see also **Figure 2a**). Levels of the housekeeping genes *GAPDH* and *ACTB* (highlighted as cluster A in **Figure 3a**) are correlated at all time points, which reflects the well known phenomenon of variation in total RNA content between cells.

At day 9 and day 16, a cluster of correlated gene expression emerges (highlighted as cluster B in **Figure 3a**) demonstrating coexpression of genes such as *SLC17A6*, *CNR1*, and *SCN9A*. It is noticeable that this cluster is anticorrelated with the group of genes that includes earlier markers such as *PAX6* and *NEUROG1* (highlighted as cluster D). This anticorrelation confirms that as cells acquire a phenotype typical of DRG neurons they lose expression of these earlier markers and also that the later *PAX6* expression described above, tends to come from a non-neuronal population rather than residual expression in the neurons themselves.

Another notable cluster forms around *PHOX2B*, *RET*, *CHAT*, and *ISL1* (highlighted as cluster C in **Figure 3a**). Expression of these genes is somewhat correlated with the expression of other neuronal genes, but also shows a distinct separate subclustering. *PHOX2B* is a transcription factor associated with the autonomic sympathetic nervous system.^19^ It is known that *RET* itself is a direct target of *PHOX2B*^20^ and that *RET* is involved in the development of cholinergic (*CHAT*^+^) sympathetic neurons;^21^ so, coexpression of these genes is not unexpected. The inclusion of *ISL1* in this subcluster is surprising however. We confirmed the presence of cells coexpressing this cluster of genes by assessing the significance of the overlap of the populations expressing each gene with the population expressing *PHOX2B*. The overlap was significant in all cases (hypergeometric, *P* < 0.05), however it should be noted that only 4 cells out of 77 assayed at day 16 expressed any detectable levels of *PHOX2B* (~5%), so this is not a source of significant heterogeneity overall.

To summarize the populations observed in the single-cell data further, we clustered the cells by the partitioning around medoids method.^22^ **Figure 3b** shows a summary of the six clusters obtained in terms of the percentage of cells within each cluster that express certain key markers. **Figure 3c** shows how the proportion of the cell population at each time point is made up of cells from each of the six clusters.

As expected, at the first two time points, the dominant cell populations fall into cluster 1, which is neuroectodermal in nature (*PAX6*^+^ and to a lesser extent *LHX2*^+^). This population steadily disappears to be replaced by cells from clusters 3 and 4. Cluster 2 cells appear at a relatively constant, but low level throughout the time course. They are generally *PAX6*^+^ or *NEUROG1*^+^, but also express *CNR1* at a low level (at least 16-fold lower than in cells belonging to clusters 5 or 6; *t*-test; *P* < 2e^−16^). The gene panel we have available does not allow a clear classification of cells in cluster 3, but cluster 4 is clearly *NEUROG1*^+^ and *ASCL1*^+^, suggesting a neural crest phenotype. The cells from clusters 5 and 6 that emerge at days 13 and 16 replace these intermediate populations in turn. Cells in clusters 5 and 6 are almost all *CNR1*^+^ (indicating neuronal identity) and in the case of cluster 6 *SCN9A*^+^ as well. The combination of these two clusters represents >50% of all cells at day 16; and cluster 6 alone represents >25%. The *PHOX2B*^+^ cells identified above are not numerous enough to form a separate cluster in this analysis.

### hPSC-sensory express a wide panel of sensory ion channels

An *in vitro* model requires cells that are functionally equivalent to their *in vivo* counterparts. In order to aid the functional characterization of hPSC-sensory, we determined their ion channel transcriptome and compared them with that of hDRGs. To date, there has been no systematic functional analysis performed on human sensory neurons due to their scarcity. hPSC-sensory, therefore, provide a unique opportunity to analyze the ion channel complement expressed in human sensory neurons and to test pharmacological molecules.

**Figure 4:**
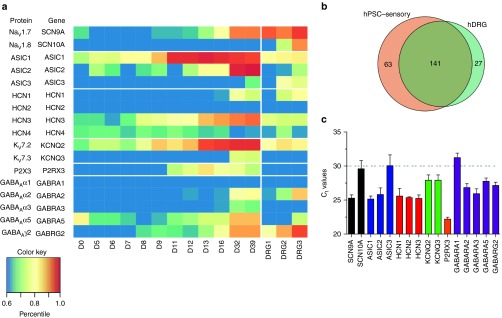
**Expression of sensory ion channel genes in human pluripotent stem cells (hPSC)-sensory during the directed differentiation time course and in hDRG samples**. (**a**) The heatmap shows the expression values of selected ion channel genes given as percentiles of the genome wide expression levels. (**b**) The overlap between the total numbers of ion channel genes expressed in hPSC-sensory and hDRG is given in the Venn diagram. (**c**) qPCR analysis confirms the expression of sensory ion channels in hPSC-sensory.

hPSC-sensory expressed 204 of the 387 ion channel encoding genes present in the human genome and 141 of the 168 ion channel genes expressed in hDRG samples (84 %; **Figure 4b**). These summary numbers suggest that hPSC-sensory should form a good model of human sensory neurons, but to confirm this, we investigated a set of specific channels involved in pain sensation.

The final set of ion channels chosen for further analysis was based on their positive expression in both hPSC-sensory and hDRG samples along with an established functional role in pain signaling in rodents. All of these ion channels are known to be expressed in rodent DRG neurons but their presence in hDRG neurons has not yet been described. **Figure 4a** shows the absolute expression profiles of these genes during differentiation and in the three hDRG samples. qPCR analysis of ion channel subunits also confirmed the expression of key sensory channels supporting the microarray transcription profile (**Figure 4c**). Generally, evidence of gene expression, as we have presented so far, can be considered necessary, but not sufficient for evidence of true function. To obtain a true functional characterization of hPSC-sensory, we performed patch-clamp electrophysiological recordings to assess the presence of particular tissue relevant ion channels. Pharmacological tools were then utilized to identify the underlying ion channel subtypes and biophysics.

### hPSC-sensory express α2/α3/γ2 containing GABA_A_R

GABA A receptors (GABA_A_R) are a family of ligand-gated ion channels and GABA_A_R modulation is the pharmacological mode of action for the widely used benzodiazepine family of drugs. GABA_A_R expression has been reported in DRG neurons from both rodents and humans^23,24,25^ with presynaptic α2-GABA_A_R forming a major site for the analgesic action of diazepam.^26^ We observed low levels of GABA_A_R subunit mRNA expression in hPSC-sensory from an early time point (α5 and γ2; day 0–8) with increased expression of these and other subunits later (α2, day 32). We did not observe α1 expression in hPSC-sensory in either the microarray or qPCR analyses with only low levels of α3 (**Figure 4a**,**c**). This pattern of expression was well matched by that of the hDRGs where γ2, α2, and α5 were observed (with no α1 or α3).

**Figure 5:**
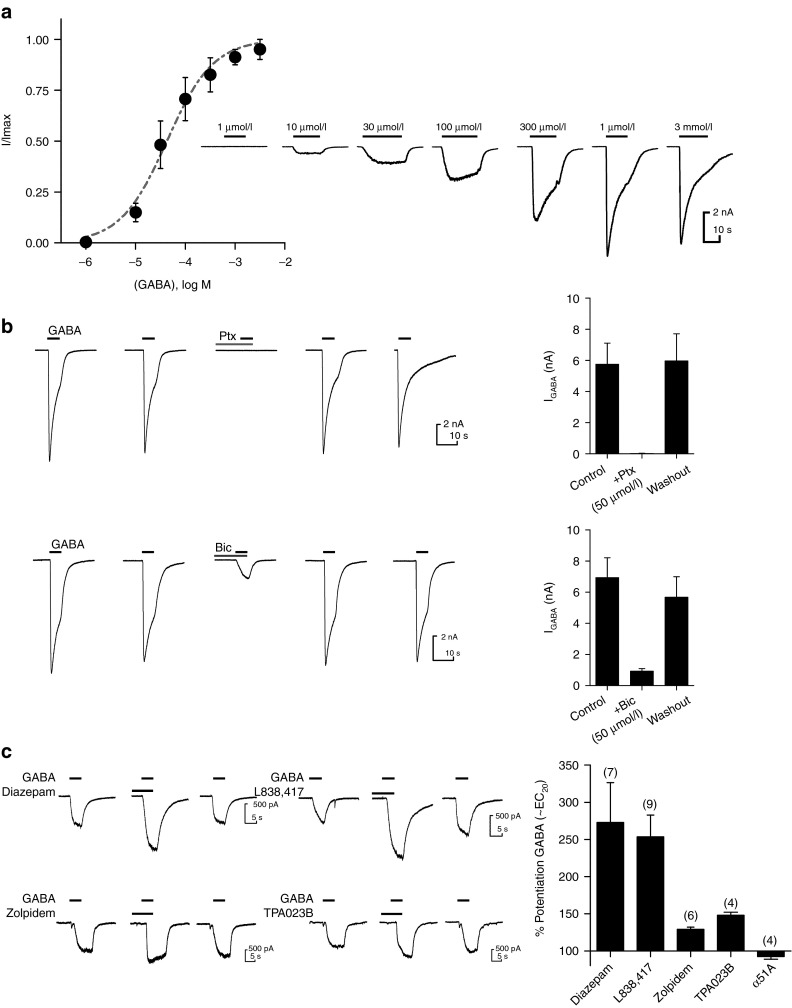
**GABA**_**A**_**receptors in human pluripotent stem cells (hPSC)-sensory contain α2, α3, γ2 subunits and exhibit sensitivity to classical GABA**_**A**_ **ligands**. (**a**) GABA-induced inward currents were observed when hPSC-sensory cells were clamped at −60 mV. Representative traces in response to increasing concentrations of GABA can be seen in right panel and concentration–response curve in left panel (EC_50_ = ~100 µmol/l). (**b**) Reproducible I_GABA_ were seen in response to an EC_100_ concentration of GABA (1 mmol/l) these currents were completely blocked by the noncompetitive pore blocker picrotoxin (control: 5.8 ± 1.4 pA/pF, picrotoxin: 50 µmol/l 0.0 ± 0.01 pA/pF, wash: 6.0 ± 1.7 pA/pF *n* = 5; top panel) and largely inhibited by the competitive ligand bicuculine (control: 6.9 ± 1.3 pA/pF, bicuculline: 50 µmol/l 0.9 ± 0.2 pA/pF, wash: 5.7 ± 1.3 pA/pF *n* = 5; bottom panel). (**c**) A submaximal concentration of GABA (EC_20_) was used to assess selective and nonselective GABA_A_R-positive allosteric modulators. Expression of γ2 was demonstrated by potentiation of *I*_GABA_ with diazepam (10 µmol/l; 273.0 ± 53.6%, *n* = 7). Potentiation was observed with the α2/α3-preferring PAMs L838, 417 (1 µmol/l; 253.6 ± 29.3%, *n* = 9) and TPA023B (1 µmol/l; 148 ± 4.2%, *n* = 4), whereas minimal potentiation was observed with the α1-prefering PAM Zolpidem (10 nmol/l; 129.2% ± 2.8, *n* = 6). α5IA had little effect at *I*_GABA_ (92.5 ± 3.6%, *n* = 4).

Consistent with this expression profile, fast GABA-induced currents (*I*_GABA_) were observed in patch-clamp recordings of hPSC-sensory (**Figure 5a**). In response to a maximal GABA concentration, *I*_GABA_ were large (5,023.3 ± 1.8 pA; *n* = 7) and rapidly desensitized. *I*_GABA_ responded in a dose-dependent manner with an EC_50_ of 100 µmol/l (**Figure 5a**). Next, we examined the pharmacological profile of *I*_GABA_. The GABA_A_R antagonists picrotoxin and bicuculline completely blocked *I*_GABA_ following a short preincubation (**Figure 5b**). In addition to these classical antagonists, we assessed subunit-selective positive allosteric modulators (PAMs) at *I*_GABA_. Diazepam is a PAM of γ2-containing GABA_A_R. Our array and qPCR expression data (**Figure 4b**,**c**) demonstrate the expression of the γ2 subunit; so, *I*_GABA_ would be predicted to be diazepam sensitive. Consistent with this expression data, *I*_GABA_ was potentiated by diazepam (273 ± 53.6%, *n* = 7; **Figure 5c**). Next we utilized α-subunit-selective PAMs to probe which α subunits are likely to be expressed by hPSC-sensory. L838, 417 (a α2/3 preferring PAM) showed robust potentiation of *I*_GABA_ (253.6 ± 29.3%, *n* = 9; **Figure 5c**) Zolpidem (a α1-preferring PAM) exhibited minimal potentiation suggesting little α1 expression (129.2 ± 2.8%, *n* = 6; **Figure 5c**), whereas TPA023B (another α2/3 preferring PAM) exhibited robust potentiation (148.0 ± 4.2%, *n* = 4; **Figure 5c**). Finally, α5IA (a α5-negative allosteric modulator) showed little to no modulation of *I*_GABA_ suggesting no α5 expression (92.5 ± 3.6%, *n* = 4, **Figure 5c**). Taken together, this functional data support the expression data above that hPSC-sensory, like hDRG, expresses GABA_A_R containing γ2, α2, and α3 subunits with little to no contribution from α1 and α5.

### hPSC-sensory express HCN1 ion channels

**Figure 6:**
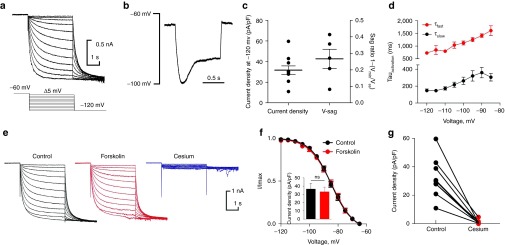
**Human pluripotent stem cells (hPSC)-sensory express a fast cAMP-insensitive *I***_**h**_ **indicative of HCN1 ion channels**. (**a**) Hyperpolarization-activated currents (*I*_h_) were observed in hPSC-sensory when cells were voltage-clamped from a *V*_h_ of −60 mV to hyperpolarizing test voltage steps from −65 to −120 mV in 5 mV increments (bottom panel). Utilizing tail current analysis (see Materials and Methods), the *V*_1/2_ of activation of *I*_h_ in hPSC-sensory was −84.7 ± 1.0 mV (*n* = 5). (**b**) Voltage relaxations (v-sag) typical of HCN-expressing neurons were observed in response to a hyperpolarizing injection in current-clamp mode (60 pA in this representative cell). (**c**) The average current density measured in voltage-clamp in response to a −120 mV voltage step was 31.8 ± 4.2 pA/pF (*n* = 10) in current-clamp mode an average of 0.3 ± 0.1 v-sag (*n* = 5) was observed in response to current injection achieving target voltages of ~−100 mV. (**d**) Activation kinetics were analyzed by fitting a double exponential to describe two activation rates (*τ*_fast_ and *τ*_slow_). *τ*_fast_ kinetic analysis demonstrate a rapidly activating *I*_h_ similar to those observed in recombinant HCN1 ion channels. (**e**) Family of *I*_h_ traces in response to voltage steps as shown in **a**. In these examples, the cell was either measured in control conditions, forskolin-treated or in the presence of the *I*_h_ blocker cesium. (**f**) Voltage-activation curves for *I*_h_ were not affected following forskolin treatment (*V*_1/2_ in control = −84.74 ± 1.0 mV and forskolin-treated = −84.0 ± 1.1 mV; *n* = 5; not significantly different). Likewise, current densities measured at −120 mV were not different between control (36.4 ± 6.8 pA/pF) and forskolin-treated (33.0 ± 5.6 pA/pF) cells. (**g**) *I*_h_ was completely blocked by a short application of the HCN channel blocker cesium (control = 31.8 ± 4.2 pA/pF and cesium = 0.9 ± 0.4 pA/pF, *n* = 10).

HCN ion channels are a family of four hyperpolarization-activated ion channels that contribute to cardiac and neuronal pace making.^27^ HCN channels regulate action potential frequency in DRG neurons with HCN1, 2, and 3 showing the most prominent expression.^28^ Our microarray data show that hPSC-sensory and hDRGs express the HCN1 and HCN3 subunits, with little to no HCN2 expression, though all three transcripts were detected by qPCR (**Figure 4**). Patch-clamp experiments show that hPSC-sensory express a hyperpolarization-activated current (*I*_h_) in response to hyperpolarizing voltage steps (**Figure 6a**,**c**) and exhibit a voltage-sag in response to hyperpolarizing current injections typical of HCN ion channel expression (**Figure 6b**,**c**).

*I*_h_ activation kinetics vary depending on the subunits incorporated in the native channel with activation kinetics in the order of HCN1<HCN2<HCN3<HCN4 where HCN1 is the fastest.^29^ In hPSC-sensory, *I*_h_ was well described by two exponential fits (*τ*_fast_ and *τ*_slow_, **Figure 6d**). This analysis showed that *I*_h_ activation was fast, typical of HCN1 expression^29^ with *τ*_fast_ = 147.4 ± 20.0 and *τ*_slow_ = 729.6 ± 47.7 msec, *n* = 9. Next, we tested whether this fast *I*_h_ is cAMP sensitive (HCN1 and 3 are cAMP-insensitive whereas HCN2 activation is accelerated by cAMP). *I*_h_ was measured in response to voltage steps in control, forskolin, and cesium conditions (**Figure 6e**). *I*_h_ activation curves were not affected by forskolin treatment nor were current densities (**Figure 6f**). These cAMP-insensitive currents were blocked by cesium in a manner typical of HCN channels. All of which demonstrates that hPSC-sensory express a fast cAMP insensitive *I*_h_ that is likely to be conducted by either HCN1 homomeric or HCN1/HCN3 heteromeric ion channels.

### hPSC-sensory express KCNQ2/3

**Figure 7:**
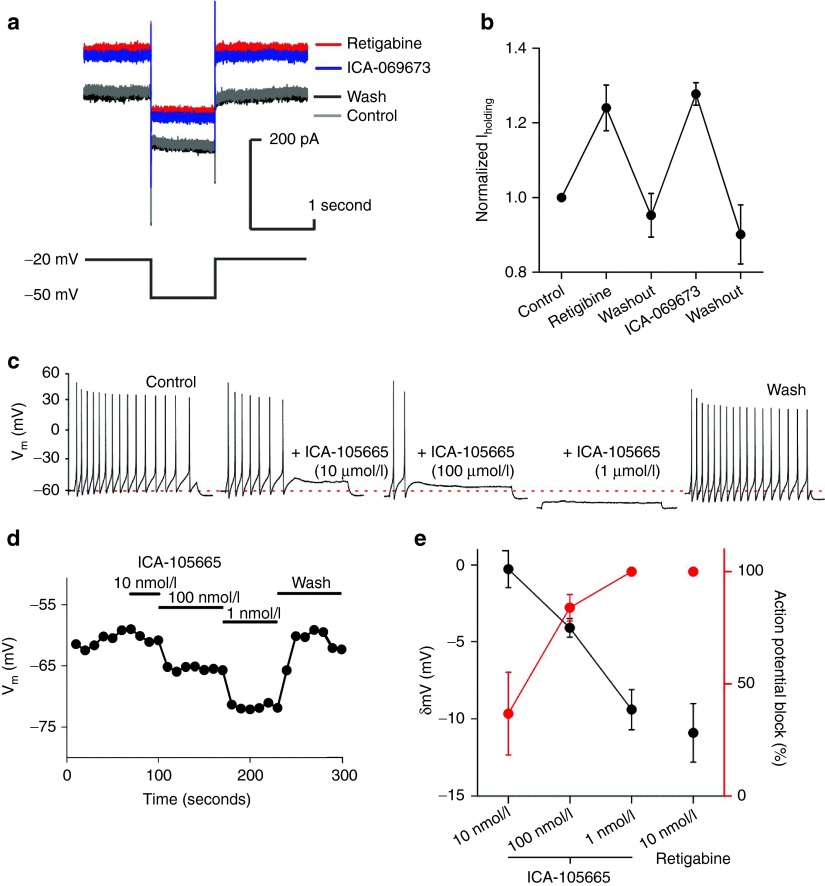
**Human pluripotent stem cell (hPSC)-sensory express KCNQ2/3 channels and a retigabine-sensitive *I***_**K(M)**_ **which contribute to resting membrane potential and regulate excitability**. (**a**) *I*_K(M)_ was identified utilizing an activation voltage-step protocol (bottom panel) a small slow current relaxation was observed when stepping to a −50 mV deactivation step resulting from *I*_K(M)_ deactivation. (**b**) Retigabine- and ICA-069673-sensitive current were observed and quantified (retigabine: 1.2 ± 0.1 normalized current, *n* = 4; wash: 1.0 ± 0.1 normalized current, *n* = 4; ICA-069673: 1.3 ± 0.01, *n* = 4; wash: 0.9 ± 0.1, *n* = 4). (**c**) KCNQ2/3 openers affect hPSC-sensory membrane potential and excitability. Representative trace of action potential trains evoked with a depolarizing current injection. The KCNQ opener ICA-105665 hyperpolarizes the membrane potential resulting in fewer evoked action potentials in a concentration-dependent manner and could be rapidly washed. The initial resting membrane of −60 mV is represented by the red dotted line. (**d**) Representative experiment demonstrating the membrane potential for ICA-105665. (**e**) Quantification showing the inverse relationship between RMP and evoked action potentials in hPSC-sensory.

KCNQ2/3 (K_v_7.2/7.3) channels are expressed in rodent DRG neurons^30^ and are key ion channels for controlling neuronal resting membrane potential (RMP). KCNQ2/3 transcripts were detected in our microarray and qPCR analyses with expression of both in hPSC-sensory cultures and KCNQ2 alone in hDRG (**Figure 4b**). We therefore examined whether KCNQ2/3 ion channels were also detected at a functional level. We utilized compounds known to be openers of KCNQ2/3 in conjunction with an inactivation protocol^30^ to examine their expression in hPSC-sensory. The pan-KCNQ opener retigabine and the KCNQ2/3 selective opener ICA-069673 (ref. 31) both increased the potassium conductance using this protocol (**Figure 7a**). Both openers were readily washable (**Figure 7b**) demonstrating hPSC-sensory express KCNQ2/3 ion channels. KCNQ2/3 ion channels have also been reported to regulate neuronal RMP; so, we investigated whether KCNQ openers affected RMP and evoked action potential firing frequency in hPSC-sensory. Both retigabine and KCNQ opener ICA-105665 (ref. 32) hyperpolarized the membrane in a reversible and blockable manner (**Figure 7c**,**d**). Consistent with RMP hyperpolarization, both KCNQ2/3 openers blocked evoked trains of action potentials in a concentration-dependent manner demonstrating their ability to suppress human nerve excitability; a correlate for pain transmission (**Figure 7c**,**e**).

### hPSC-sensory express heteromeric ASICs

**Figure 8:**
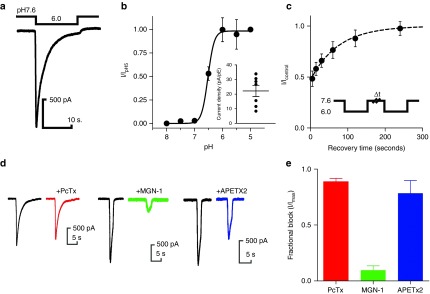
**Human pluripotent stem cells (hPSC)-sensory express ASIC1/2 heteromers and ASIC3 homomeric ion channels**. (**a**) Representative trace of an inward current in response to a pH drop from physiological (pH 7.4) to acidic (pH 6.0) conditions. (**b**) Concentration–response curve for hPSC-sensory expressed ASICs. Similarly to recombinantly expressed ASICs, the Hill slope is very steep (Hill slope = 3.9) with an IC_50_ = pH 6.5 (*n* = 7). Inset: non-normalized peak current densities observed at pH 6.0 (22.2 ± 1.7 pA/pF, *n* = 7). (**c**) *I*_pH_ recovery from desensitization was measured by two pH 6.0 drops separated by a test recovery period. Currents were fully recovered with a recovery period between 120 and 240 seconds (*τ*_recovery_ = 111.1 ± 19.8 seconds). (**d**) Representative traces to pH6 before (black traces) and after subunit-selective blockers psalmotoxin (ASIC1a left panel), mambalgin-1 (ASIC1a/b 2a/b middle panel), APETx2 (ASIC3 right panel). (**e**) Quantification of toxin inhibition as shown in **d**. Psalmotoxin exhibited limited to no inhibition of *I*_pH_ in hPSC-sensory (0.9 ± 0.01 fraction of control, *n* = 11), whereas mambalgin-1 showed large inhibition demonstrating the presence of heteromeric ASICs (0.1 ± 0.01 fraction of control, *n* = 4). APETx2 showed small block (0.8 ± 0.1 fraction of control, *n* = 4) suggesting low levels of ASIC3 homomers.

Acid-sensing ion channels (ASICs) are expressed throughout the pain pathway and have been reported in rodent DRG neurons where they are proposed to mediate inflammatory pain.^33,34^ Our microarray and qPCR data demonstrate strong expression of ASIC1 and 2 and low levels of ASIC3, a comparable pattern to hDRG (**Figure 4**). We examined whether hPSC-sensory expressed ASICs by patch-clamp experiments by applying a pH drop from physiological (pH 7.6) to acidic (pH 6.0) conditions. This pH drop induced a fast transient inward current *I*_pH_ (**Figure 8a**). *I*_pH_ exhibited a steep pH dose–response curve with maximal activation achieved between pH 6.5 and pH 6.0 (Hill slope ~4.0, pH_50_ = pH 6.5 ± 0.1, *n* = 6; **Figure 8b**). Recovery rates from inactivation were investigated by separating two test pH drops by increasing recovery periods (**Figure 8c**). Recovery from pH inactivation was achieved after ~200 seconds.

Using this information, we probed the subunit composition of *I*_pH_ by utilizing ASIC-subunit selective toxins. First, we found that the ASIC1a blocker psalmotoxin did not affect *I*_pH_. Mambalgin-1, a recently identified blocker of heteromeric^35^ ASICs, strongly blocked *I*_pH_ demonstrating that this current is composed mostly of heteromeric ASICs. The small proportion remaining unblocked by mambalgin-1 was accounted for by blocking with APETx2 suggesting low levels of ASIC3 homomers.

## Discussion

Despite great interest in sensory and pain biology and a clear unmet medical need, the vast majority of research in this area is conducted on rodent cellular and animal models. The use of human tissue tends to be restricted to molecular and biochemical assays and even then cellular material is hard to acquire. With these factors in mind, the protocols designed for reprogramming pluripotent stem cells into disease relevant tissues is a crucial endeavor. The establishment and use of these protocols will increase our confidence that results will be clinically translatable and will result in the reduced use of animals in biomedical research.

Here, we focus on an in-depth molecular, cellular, and functional characterization of the hPSC-sensory at key points through directed differentiation. We found that our hPSC-sensory protocol was capable of effectively neuralizing pluripotent cells, driving them through a neuroectodermal fate followed by neural crest and the formation of a distinct nociceptor phenotype; a process that broadly recapitulates the *in vivo* developmental process.^18^ Importantly, we extend our previous work^9^ by assessing the genome-wide mRNA transcript profile of our cells at later time points than previously and by comparing those profiles directly to those of hDRG—the relevant *in vivo* tissue. These analyses clearly show that the mRNA expression profile of hPSC-sensory are highly similar to cells from hDRG and that this expression profile is achieved after 2–3 weeks in growth factor media, after which it appears not to change dramatically. Inspection of key functional categories of genes such as ion channels shows that the patterns of expression of these genes in hPSC-sensory are similar to hDRG. Key sensory ion channel genes (SCN9A, SCN10A, TRPV1, and P2X3) are expressed in hPSC-sensory and human DRG. Unexpectedly, we detected SCN3A (Nav1.3) which is typically reported to be silenced during development and upregulated following injury. This gene, and others, may demonstrate that hPSC-sensory remain juvenile neurons or that they are injured during the RNA isolation process. In contrast, the residual differences we see in gene expression between hPSC-sensory and hDRGs are largely accounted for by expression within the hDRG of immune regulatory genes and genes involved in the formation of Schwann cell–derived fatty myelin (**Supplementary Table S1**). The presence of these genes within hDRG and their absence in hPSC-sensory is expected given the extracted postmortem DRG used in this study and is desirable in terms of hPSC-sensory providing a more homogenous source of sensory neurons uncontaminated with other cell types.

We also provide a characterization of the differentiating cells and final neurons at the single-cell level. Single-cell expression analyses are fast becoming a crucial tool in understanding the underlying dynamics of mRNA expression and cellular differentiation.^11,36^ Key to this work is the direct measurement it provides of the homogeneity of the culture, which can be masked in population level measurements. Our data show that ~70% of cells in the final culture are neuronal as evidenced by expression of *CNR1* and that ~70% of the neurons have a nociceptor phenotype as evidenced by *SCN9A* expression. The final time point for which we have single-cell expression data (day 16) is not late enough to observe *RUNX1*^+^ cells, but the microarray data demonstrate that expression of this sensory neuron transcription factor is seen at significant levels later on in the time course confirming that the full maturation of these cells in growth factor media takes at least 2–3 weeks.

The neuronal cells we observe at day 16 emerge from almost 100% *PAX6*^+^ and 75% *NEUROG1*^+^ populations representing neuroectodermal precursors and neural crest, respectively. A small population of *PHOX2B*^+^ neurons (around 5% of the total) are also present in our final culture, suggesting that some neurons may have differentiated down an autonomic sympathetic lineage. Sensory neurons and sympathetic neurons both derive from neural crest precursors and respond to many of the same growth factors;^37^ so, it is not surprising that we observe these cells in our cultures. Further work to optimize culture conditions later in the protocol may allow the proportion of sympathetic neurons to be reduced (or increased) further as desired.

Interestingly, it is evident from both the microarray and the single-cell qPCR data that while expression of the neural crest markers disappears once the neural population establishes itself, the neuroectodermal markers, *PAX6* and *LHX2*, remain expressed in a subset of cells. The precise identity of these potentially contaminating cells remains unclear as techniques such as qPCR are limited in the number of marker genes that can be assayed for simultaneously, and these cells do not express significant amounts of any of the other markers. The same is true of ~30% of cells present at the day 9 intermediate time point suggesting that these contaminating cells may emerge from a non-neural crest like population. Further characterization of these populations will require genome-wide single-cell profiling methods such as single-cell RNA-Seq.^38,39^

An important caveat to these molecular and cellular studies is that expression of mRNA transcript is a necessary, but not sufficient condition for the presence of functional proteins and therefore cells with practical use for disease modeling. mRNA must be translated and proteins such as ion channels trafficked to the correct cellular location, while cells must themselves assume the correct morphology before truly functional neurons form. By inspection of the expression data for mature hPSC-sensory and hDRG, we hypothesized the presence of particular ion channels and subtypes and then tested those hypotheses through rigorous electrophysiological and pharmacological assays. These assays support the conclusion that hPSC-sensory are in many respects functionally equivalent to hDRG neurons and therefore form a practical alternative to rodent cells for pharmacological investigation of pain disorders.

GABA_A_R, for example, are well-characterized ligand-gated ion channels with a role in pain processing at the level of the spinal dorsal horn.^26^ GABA_A_R have reported expression in both rodent and human DRG neurons^23,24^ where they exert their analgesic effect. In cultured human dorsal root ganglia neurons, GABA_A_ α subunits have an expression profile of α2≥α3>α5>>α4/α1 (ref. 23), which is comparable to the microarray and qPCR data described here for hPSC-sensory and hDRG with high α2, α3, and α5 expression and undetectable α1 expression. Interestingly, patch-clamp data have also been acquired from cultured hDRG neurons with a reported GABA EC_50_ of 111 µmol/l^24^ similar to those described here for hPSC-sensory. Our microarray data detected high expression of the γ2 subunit, which agrees with our patch-clamp data demonstrating potentiation with diazepam. Subtype selective PAMs support the interpretation that hPSC-sensory have high α2/α3 expression (potentiation with TPA023B and L838, 417) with low α1 expression (no potentiation by zolpidem). Interestingly, L838,417, which is usually described as a submaximal positive allosteric modulator^40^ exhibited full potentiation (when compared to diazepam) of hPSC-sensory *I*_GABA_ when compared to diazepam possibly demonstrating a unique characteristic of native human GABA_A_R.

HCN ion channels are a pain target of great interest with both HCN1 and HCN2 isoforms implicated.^28,41,42^ Our microarray data suggest a higher expression of HCN1 in both hPSC-sensory and hDRG. Similarly, hPSC-sensory expressed a cAMP-insensitive fast *I*_h_ comparable to HCN1 ion channels expressed in recombinant systems^29,43^ and large rodent DRG neurons.^44^ Whether the cAMP-insensitivity in hPSC-sensory *I*_h_ is indicative of expression of HCN1 homomers or whether insensitive heteromers are also expressed is as yet unclear.

KCNQ ion channels (K_v_7.2/7.3) have been reported in rodent DRGs^30^ where they mediate analgesic effects by modulating neuronal RMP.^45,46^ Although KCNQ channels have been reported in rodent DRG neurons there have been no equivalent reports in hDRG neurons. Here, we found that similarly to rodent DRG neurons hPSC-sensory expressed KCNQ2/3 ion channels, which were modulated by the pan-KCNQ opener retigabine and the KCNQ2/3 openers ICA-069673 and ICA-105665. The RMP of hPSC-sensory was strongly modulated by these KCNQ2/3 openers with excitability being inhibited in a dose dependent manner supporting the role for these channels as pain targets and hPSC-sensory as tools for drug testing.

Sensing small changes in extracellular acidosis in peripheral tissue is achieved by the expression of ASICs in the axons of DRG neurons. It is unclear which of the five functional ASIC subunits (ASIC1a, 1b, 2a, 2b, 3) are expressed in hDRG sensory neurons, but in rodents they are all expressed to varying levels. We were able to probe *I*_pH_ in hPSC-sensory utilizing toxins with different subtype selectivity. We found that the homomeric-selective toxin psalmotoxin (ASIC1a)^47^ did not block acid-induced currents in hPSC-sensory demonstrating a lack of ASIC1a homomer expression. Supporting this idea was the fact that mambalgin-1, a recently identified blocker of heteromeric ASICs,^35^ blocked *I*_pH_ in hPSC-sensory. The remaining portion unaffected by mambalgin-1 (~10%) appears to be accounted for by the expression of ASIC3, which was blocked by APETx2 (ref. 48). While it is known that ASIC responses from rodent DRG are heterogeneous in nature,^33,49^ suggesting a mixed population of ASIC subunit expression; this has not been investigated previously in hDRG neurons.

In this work, we demonstrate how one can produce functionally differentiated human sensory neurons, suitable for disease modeling and pharmacological investigation, from pluripotent stem cells. We also show that an in-depth molecular characterization of both the pluripotent cells and the process of differentiation provide new insights into questions of cellular memory, *in vitro* development and physiology.

## Materials and Methods

***Cells and culture conditions.*** hES cells (Shef1) were cultured on hES-qualified Matrigel (BD Biosciences, San Jose, CA) as single cells at 6–12,000 cells/cm^2^. mTesr1 (StemCell Technologies, Vancouver, Canada) media was changed daily. Cells were passaged following a 1-hour preincubation with ROCK inhibitor (Sigma, Buchs, Switzerland), washed with phosphate-buffered saline (PBS) (−/−) and then TrypLE Select (Life Tech, Gent, Belgium) used to dissociate before replating onto fresh hES qualified matrigel. Human dorsal root ganglia were obtained from the Netherland Brain Bank (Amsterdam, The Netherlands).

***Neural induction.*** hES cell cultures were dissociated as above but replated onto growth factor–reduced matrigel (BD) at 100,000 cells/cm^2^ (unless otherwise stated) in TesR2 (StemCell Technologies) with ROCK inhibitor (Y-27632; Sigma). After 48 hours, the media was changed to DMEM KO ES, Knockout Serum Replacement Xeno-Free, 1× NEAA, 1× Glutamax, 0.01 mmol/l β-mercaptoethanol with the small molecule inhibitors LDN193189 (1 µmol/l) and SB-431542 (10 µmol/l) to drive anterior neuroectoderm specification. On day 5, CHIR99021 (GSK-3β inhibitor), DAPT (secretase inhibitor), and SU5402 (FGF/PDGF inhibitor) were added at a final concentration of 10 µmol/l. On day 10, the media was changed to neural growth media of Dulbecco's modified Eagle medium (DMEM)-F12, 10% fetal bovine serum with 10 ng/ml brain-derived neurotrophic factor, glial derived neurotrophic factor, neuronal growth factor, NT-3, and 200 µmol/l ascorbic acid. On day 14, the cells were treated with 0.01 mg/ml mitomycin C for 2 hours to reduce the non-neuronal population before feeding with the neural growth media. The neural growth media was then replaced every 3–4 days.

***Single-cell quantitative real-time polymerase chain reaction (qRT-PCR).*** Cells were treated with TrypLE (Invitrogen, Gent, Belgium) for 15 minutes (days 5, 7, and 9) or collagenase II (Invitrogen) for 25 minutes (days 13 and 16) to remove cells from the surface of the wells. Enzyme was neutralized with knockout DMEM (Invitrogen) containing knockout serum replacement (Invitrogen) and gently triturated to create a single-cell suspension. Cells were centrifuged at 1,300 rpm for 3 minutes then resuspended in PBS containing 2% bovine serum albumin. Cells were passed through a cell strainer before using a flow cytometer (Beckman Coulter MoFlo; Beckman, Wycombe, UK) to sort single live cells into wells of a 96-well PCR plate containing preamplification mastermix (5-µl Cellsdirect 2× reaction buffer (Invitrogen), 2.5-µl primer/probe mix, 1.2-µl TE low EDTA (Applichem, Darmstadt, Germany), 0.2-µl Superscript III Reverse Transcriptase/Platinum Taq mix (Invitrogen), 0.1-µl SUPERase-in ((Ambion, Austin, TX) per well). Preamplification was carried out under the following incubation conditions: 50 °C for 30 minutes, 95 °C for 2 minutes, then 24 cycles of (95 °C for 15 seconds, 60 °C for 4 minutes). The cDNA product was diluted 1:5 in TE low EDTA then run on a Fluidigm BioMark 96x96 Dynamic Array against 48 Taqman assays in duplicate. Data were analyzed using the Fluidigm Real-Time PCR Analysis software.

***RNA extraction and qPCR.*** Cells were washed with PBS (−/−), lysed using buffer RLT with 1% β-mercaptoethanol (Qiagen, Hilden, Germany) and stored at −80 °C. Lysates were thawed on ice and homogenized with a QIAShredder (QIAgen). RNA was extracted using the RNeasy Micro kit (QIAgen), according to the manufacturer's protocol and including on column DNase digestion. The RNA was reverse transcribed to cDNA using the Applied Biosystems (Carlsbad, CA) high capacity RNA-to-cDNA kit, with up to 1-µg RNA per reaction. Each 20-µl cDNA reaction was diluted to 200 µl with water and qPCR run on Taqman Low Density Arrays containing primer probe sets for each gene assay in duplicate (assay numbers in **Supplementary Table S2**) (Applied Biosystems). These were cycled on an Applied Biosystems 7900HT.

***Microarray and single-cell qPCR data analysis.*** cDNA extracted from hES-derived sensory neurons and hDRG were hybridized to Illumina HT12-v4 BeadArrays according to the manufacturers' instructions by Aros (Aarhus, Denmark). Probe level data were quantile normalized and log base2 transformed using R/Bioconductor^50^ and the beadarray package.^51^ Probes detected in less than three samples (Illumina detection *P* < 0.05) were excluded from downstream analysis. PCA, clustering, differential expression, visualization, and gene-set enrichment assay analysis were performed using R/Bioconductor and the pcaMethods,^52^ limma,^53^ and Gviz^54^ packages.

For the purposes of clustering, undetectable expression values in the single-cell qPCR data were replaced by values 10 times smaller than the lowest detected expression level for a given gene. Correlation between genes was calculated using the Pearson method. Clustering of cells was by the partitioning around medoids method implemented in the cluster^22^ package for R. The optimum number of clusters was determined subjectively.

Ion channel genes expressed in hDRG were defined as those detected (Illumina detection, *P* < 0.01) in two or more of the hDRG samples.

***Coverslip replating for functional analyses.*** Cells were washed with PBS (−/−) before an accutase dissociation. Once detached, the cells were centrifuged (100 g and 3 minutes) washed and centrifuged again. They were resuspended in neural growth media and triturated with decreasing size glass pipettes before being passed through a 70-µm cell strainer. The cells were once more centrifuged before resuspension into neural growth media and then onto a precoated growth factor–reduced glass coverslip (PDL or PDL-laminin).

***Electrophysiological recordings.*** All recordings were performed on fully differentiated hPSC-sensory (typically 4 weeks postgrowth factor addition) within 1–2 days of cellular dissociation at room temperature. Patch-clamp recordings were made using a Multiclamp 700A amplifier and digitized by a Digidata 2000. Glass borosilicate pipettes were used with typical *R*_pip_ of 2–5 MΩ resulting in *R*_series_ < 10 MΩ.

Unless otherwise stated, cells were recorded in extracellular solution containing (in mmol/l) NaCl (140), KCl (4), CaCl2 (1.8), MgCl2 (1), HEPES (10), glucose (5) pH was adjusted to 7.4 with NaOH (5 mol/l). Pipettes were filled with intracellular solutions containing (in mmol/l) KCl (140), MgCl2 (1.6), MgATP (2.5), NaGTP (0.5), EGTA (2), HEPES (10); pH was adjusted to 7.3

*KCNQ.* Voltage-clamp recordings were performed using an activation protocol as reported by^30^ in which cells are clamped at an activating potential of −20 mV and then step to hyperpolarizing potentials (−20 to −110 mV) to deactivate KCNQ channels and measuring the deactivating tail currents. Second, KCNQ channel expression was assessed by their affect upon RMP, Current clamp mode was used to assess RMP and frequency of evoked action potentials (see figure legends for current injection levels).

*HCN.* HCN ion channels were activated by hyperpolarizing of the membrane to voltages more negative than −60 mV. *I*_*h*_ was activated by holding cells at −60 mV followed by a prepulse potential to voltages between −120 and −65 (at 5 mV increments) for 4 seconds. followed by a test potential at −120 mV to assess tail currents. Activation curves were best described by a modified Boltzmann equation below.

*I*_h_ = bottom + (top-bottom)/(1+10^((LogIC50-voltage).HillSlope))

Activation kinetics were best described by a Levenberg–Marquert fit with two exponentials resulting in time constants τ_fast_ and τ_slow_ time constants.

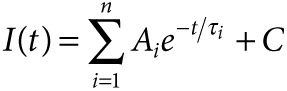


Where *I* is the current at time *t*. *A*_i_ is the initial current level for *τ*_fast_ and *τ*_slow_ and *C* is the current at steady state.

Cells were injected with varying hyperpolarizing current injections in current-clamp mode for 2 seconds. The target membrane potential was −100 mV (a voltage at which HCN channels are maximally active); the rebound potential (or voltage-sag) can then be quantified by the equation below.

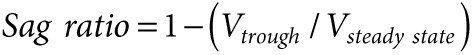


*ASICs.* ASICs were activated by pH drops from physiological pH (pH 7.6) to acidic pHs of <pH 6.5. Recordings were performed at −60 mV. Extracellular solutions (in mmol/l) 140 NaCl (140), KCl (5), CaCl_2_ (2), MgCl_2_ (1), HEPES (5), MOPS (5), D-glucose (10) pH adjusted with KOH or HCL. Intracellular solutions were as above except KCl was replaced with CsCl.

*GABAAR.* Intracellular solutions were as above except KCl was replaced with CsCl.

*Na_v_s.* Cells were voltage clamped at −120 mV and whole-cell sodium current recorded in response to a 20 ms, 0 mV activation step occurring 100 ms after 5 seconds, −75 mV inactivating prepulse (repeated every 15 seconds). Patch pipettes were filled with (in mmol/l): 135 CsF, 10 CsCl, 5 NaCl, 10 HEPES, 5 EGTA, 2 MgATP (pH 7.3, 290 mOsm).

***Drugs.*** Retigabine, ICA-105665, and Mambalgin-1 were synthesized in house. GABA, XE-991, ICA-069673 were purchased from Tocris (Bristol, UK). Forskolin and A-803467 were purchased from Sigma.

**SUPPLEMENTARY MATERIAL** **Figure S1.** Expression of key marker genes at single cell resolution measured by qPCR. **Figure S2.** Gene expression and electrophysiological comparison of two directed differentiation protocols. **Table S1.** Full gene set enrichment analysis. **Table S2.** Ion channel primers used in qPCR. **Table S3.** Summary of results comparing two directed differentiation protocols.

## Supplementary Material

Supplementary Figure S1 Expression of key marker genes at single cell resolution measured by qPCR.

Supplementary Figure S2 Gene expression and electrophysiological comparison of two directed differentiation protocols.

Supplementary Table S1 Full gene set enrichment analysis.

Supplementary Table S2 Ion channel primers used in qPCR.

Supplementary Table S3 Summary of results comparing two directed differentiation protocols.
