# Supplementary figures and images for: Characterizing Human Stem Cell–derived Sensory Neurons at the Single-cell Level Reveals Their Ion Channel Expression and Utility in Pain Research
Source: Mol Ther. 2014 Jun 17;22(8):1530–43. doi: 10.1038/mt.2014.86 (PMC4435594; doi:10.1038/mt.2014.86)

Color Key

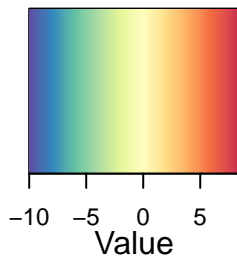

1

2

3

4

5

6

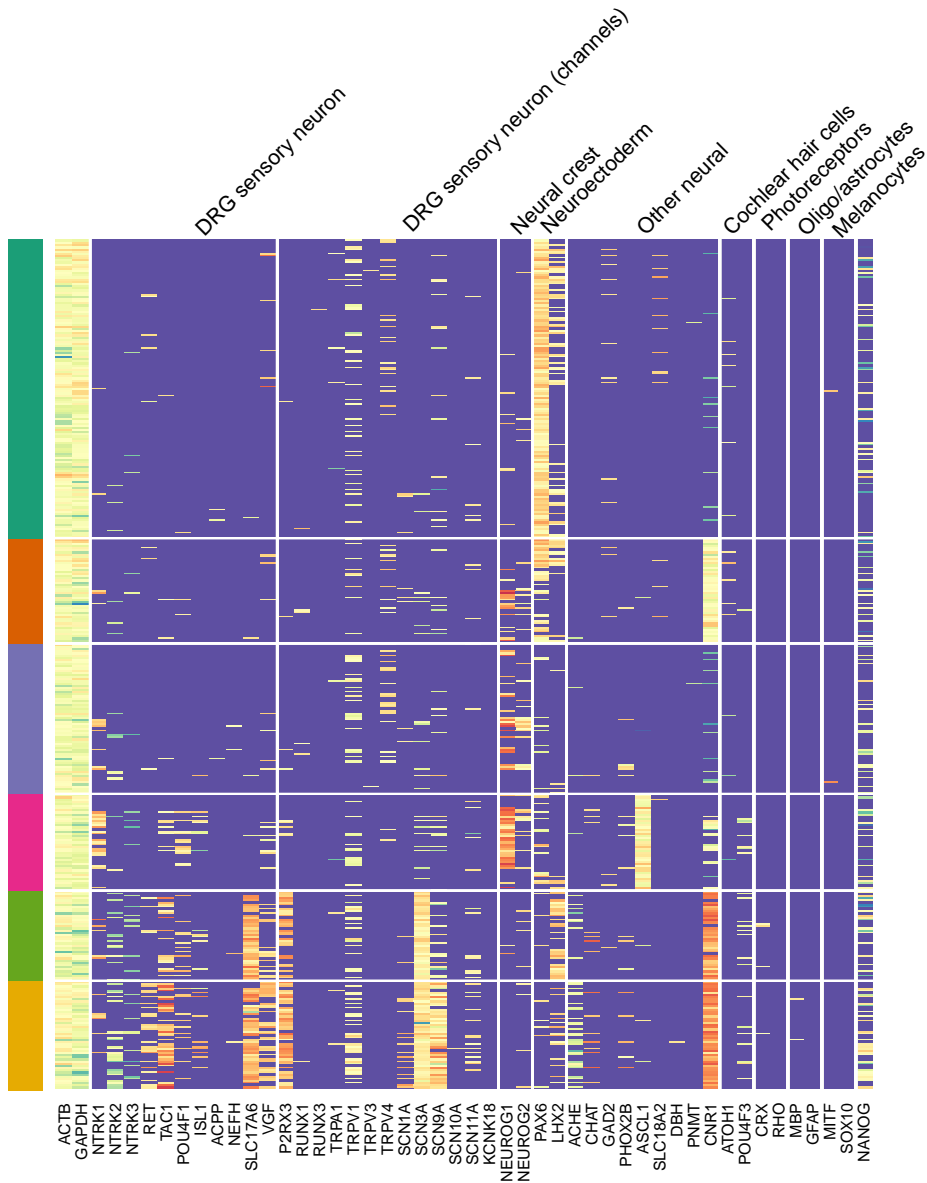

Supplement: Supplementary Figure S1 — Expression of key marker genes at single cell resolution measured by qPCR. [file mt201486x1.pdf]
