# Supplementary material for: Characterizing Human Stem Cell–derived Sensory Neurons at the Single-cell Level Reveals Their Ion Channel Expression and Utility in Pain Research
Source: Mol Ther. 2014 Jun 17;22(8):1530–43. doi: 10.1038/mt.2014.86 (PMC4435594; doi:10.1038/mt.2014.86)
Supplement: Supplementary Table S1 — Full gene set enrichment analysis. [file mt201486x3.pdf]

	NGenes	Correlation	Down	Up	TwoSided	Description
GO:0034367	26	-0.023923	0.99999972	2.81E-07	5.62E-07	macromolecular complex remodeling
GO:0034368	26	-0.023923	0.99999972	2.81E-07	5.62E-07	protein-lipid complex remodeling
GO:0034369	26	-0.023923	0.99999972	2.81E-07	5.62E-07	plasma lipoprotein particle remodeling
GO:0050778	411	0.00272256	0.99999915	8.54E-07	1.71E-06	positive regulation of immune respons
GO:0019911	8	0.00673531	0.99999919	8.12E-07	1.62E-06	structural constituent of myelin sheath
GO:0071825	31	-0.0176667	0.99999856	1.44E-06	2.89E-06	protein-lipid complex subunit organiza
GO:0071827	31	-0.0176667	0.99999856	1.44E-06	2.89E-06	plasma lipoprotein particle organizatio
GO:0032934	38	-0.0059964	0.9999989	1.10E-06	2.21E-06	sterol binding
GO:0001818	158	0.00897397	0.99999791	2.09E-06	4.18E-06	negative regulation of cytokine produc
GO:0002253	349	0.00280065	0.99999469	5.31E-06	1.06E-05	activation of immune response
GO:0002643	8	0.00709831	0.99999497	5.03E-06	1.01E-05	regulation of tolerance induction
GO:0006069	13	0.01207687	0.99999265	7.35E-06	1.47E-05	ethanol oxidation
GO:0070723	21	-0.0128517	0.99999416	5.84E-06	1.17E-05	response to cholesterol
GO:0072376	62	0.04082006	0.9999921	7.90E-06	1.58E-05	protein activation cascade
GO:0005496	75	0.00345277	0.99999239	7.61E-06	1.52E-05	steroid binding
GO:0015485	28	0.00259501	0.9999927	7.30E-06	1.46E-05	cholesterol binding
GO:0032395	11	0.05347715	0.99999293	7.07E-06	1.41E-05	MHC class II receptor activity
GO:0002517	6	0.03413239	0.9999887	1.13E-05	2.26E-05	T cell tolerance induction
GO:0002664	6	0.03413239	0.9999887	1.13E-05	2.26E-05	regulation of T cell tolerance induction
GO:0048251	7	-0.0418625	0.99998856	1.14E-05	2.29E-05	elastic fiber assembly
GO:0045940	25	-0.0145552	0.99998618	1.38E-05	2.76E-05	positive regulation of steroid metaboli
GO:0006959	99	0.02695445	0.99998547	1.45E-05	2.91E-05	humoral immune response
GO:0006956	42	0.06221363	0.99998314	1.69E-05	3.37E-05	complement activation
GO:0034439	3	-0.2745478	0.99998259	1.74E-05	3.48E-05	lipoprotein lipid oxidation
GO:0060587	3	-0.2745478	0.99998259	1.74E-05	3.48E-05	regulation of lipoprotein lipid oxidation
GO:0042613	17	0.14198888	0.99998212	1.79E-05	3.58E-05	MHC class II protein complex
GO:0002645	7	0.00865535	0.99997875	2.13E-05	4.25E-05	positive regulation of tolerance inducti
GO:0010889	9	-0.0452013	0.99997884	2.12E-05	4.23E-05	regulation of sequestering of triglyceri
GO:0002455	45	0.03031958	0.99997167	2.83E-05	5.67E-05	humoral immune response mediated b immunoglobulin
GO:0006067	17	0.01835756	0.99997231	2.77E-05	5.54E-05	ethanol metabolic process
GO:0032528	15	-0.0296959	0.99997304	2.70E-05	5.39E-05	microvillus organization
GO:0033344	40	-0.0060522	0.999972	2.80E-05	5.60E-05	cholesterol efflux
GO:0045058	26	0.00867712	0.99997514	2.49E-05	4.97E-05	T cell selection
GO:0045834	117	0.00547146	0.9999709	2.91E-05	5.82E-05	positive regulation of lipid metabolic p
GO:0060333	109	0.02810905	0.99997644	2.36E-05	4.71E-05	interferon-gamma-mediated signaling
GO:2000341	12	-0.0104583	0.9999745	2.55E-05	5.10E-05	regulation of chemokine (C-X-C motif)
GO:0044548	13	-0.0349064	0.99997629	2.37E-05	4.74E-05	S100 protein binding
GO:0001766	4	-0.1426835	0.99996776	3.22E-05	6.45E-05	membrane raft polarization
GO:0031580	4	-0.1426835	0.99996776	3.22E-05	6.45E-05	membrane raft distribution
GO:0042611	38	0.12100503	0.99996683	3.32E-05	6.63E-05	MHC protein complex
GO:0016045	15	-0.0213481	0.99996509	3.49E-05	6.98E-05	detection of bacterium
GO:0085029	14	0.0102479	0.99996357	3.64E-05	7.29E-05	extracellular matrix assembly
