# Supplementary material for: Characterizing Human Stem Cell–derived Sensory Neurons at the Single-cell Level Reveals Their Ion Channel Expression and Utility in Pain Research
Source: Mol Ther. 2014 Jun 17;22(8):1530–43. doi: 10.1038/mt.2014.86 (PMC4435594; doi:10.1038/mt.2014.86)
Supplement: Supplementary Table S2 — Ion channel primers used in qPCR. [file mt201486x4.pdf]

Gene Name	Taqman Assay ID (Life Technologies) or primer sequence
SCN9A	Hs00161567_m1
SCN10A	Hs01045137_m1
ASIC1	Hs00952807_m1
ASIC2	Hs00153756_m1
ASIC3	Hs00245097_m1
HCN1	Hs01085412_m1
HCN2	Hs00606903_m1
KCNQ2	Hs00542513_m1
KCNQ3	Hs00189619_m1
P2RX3	Hs01125554_m1
GABRA1	Hs00168058_m1
GABRA2	Hs00168069_m1
GABRA3	Hs00168073_m1
GABRA5	Hs00181291_m1
GABRG2	Hs00168093_m1
IPO8 (reference)	Hs00183533_m1
POU5F1	Hs00742896_s1
HCN3 (Sybr green)	F: CCG ATG GAT CCT ACT TTG GGG
	R: GAA TTC TTC TTG CCG ATG CGG
