# Supplementary material for: Characterizing Human Stem Cell–derived Sensory Neurons at the Single-cell Level Reveals Their Ion Channel Expression and Utility in Pain Research
Source: Mol Ther. 2014 Jun 17;22(8):1530–43. doi: 10.1038/mt.2014.86 (PMC4435594; doi:10.1038/mt.2014.86)
Supplement: Supplementary Table S3 — Summary of results comparing two directed differentiation protocols. [file mt201486x5.pdf]

Time	ICC(Peripherin, NeuN, Islet1, Brn3a)		Calciun			
	Young *et al.*	Studer *et al.*	Young *et al.*			
			ATP	veratridine	capsaicin	KCL
**D5**						
**D10**						
**1w post GF**	v	v	x	x	x	v
**2w post GF**	v	v	v	v	x	v
**3w post GF**	v	v	v	v	x	v
**4w post GF**	v	v	v	v	x	v
**5w post GF**	v	v	v	v	x	v
**6w post GF**	v	v	v	v	v	v
**7w post GF**	v	v				
**8w post GF**			v	v	v	v

A comparison of the protocol described here and in Chambers *et al.* (2012)

Key
X = no response
V = response seen

ICC = immunocytochemistry
ATP (P2X3R agonist)
veratridine (voltage-gated sodium channel agonist)
capsaicin (TRPV1 agonist)
KCl (non-selective neuronal activator)

n imaging				Electrophysiology	
Studer *et al.*				Young *et al.*	Studer *et al.*
ATP	veratridine	capsaicin	KCL	I_{Na}	I_{Na}
v	x	x	v		
v	v	x	v	v	v
v	v	x	v	v	v
v	v	x	v	v	v
v	v	x	v	v	v
v	v	v	v	v	v
				v	v
v	v	v	v	v	v
