# Supplementary material for: Characterizing Human Stem Cell–derived Sensory Neurons at the Single-cell Level Reveals Their Ion Channel Expression and Utility in Pain Research
Source: Mol Ther. 2014 Jun 17;22(8):1530–43. doi: 10.1038/mt.2014.86 (PMC4435594; doi:10.1038/mt.2014.86)
Supplement: Supplementary Figure S2 — Gene expression and electrophysiological comparison of two directed differentiation protocols. [file mt201486x2.pdf]

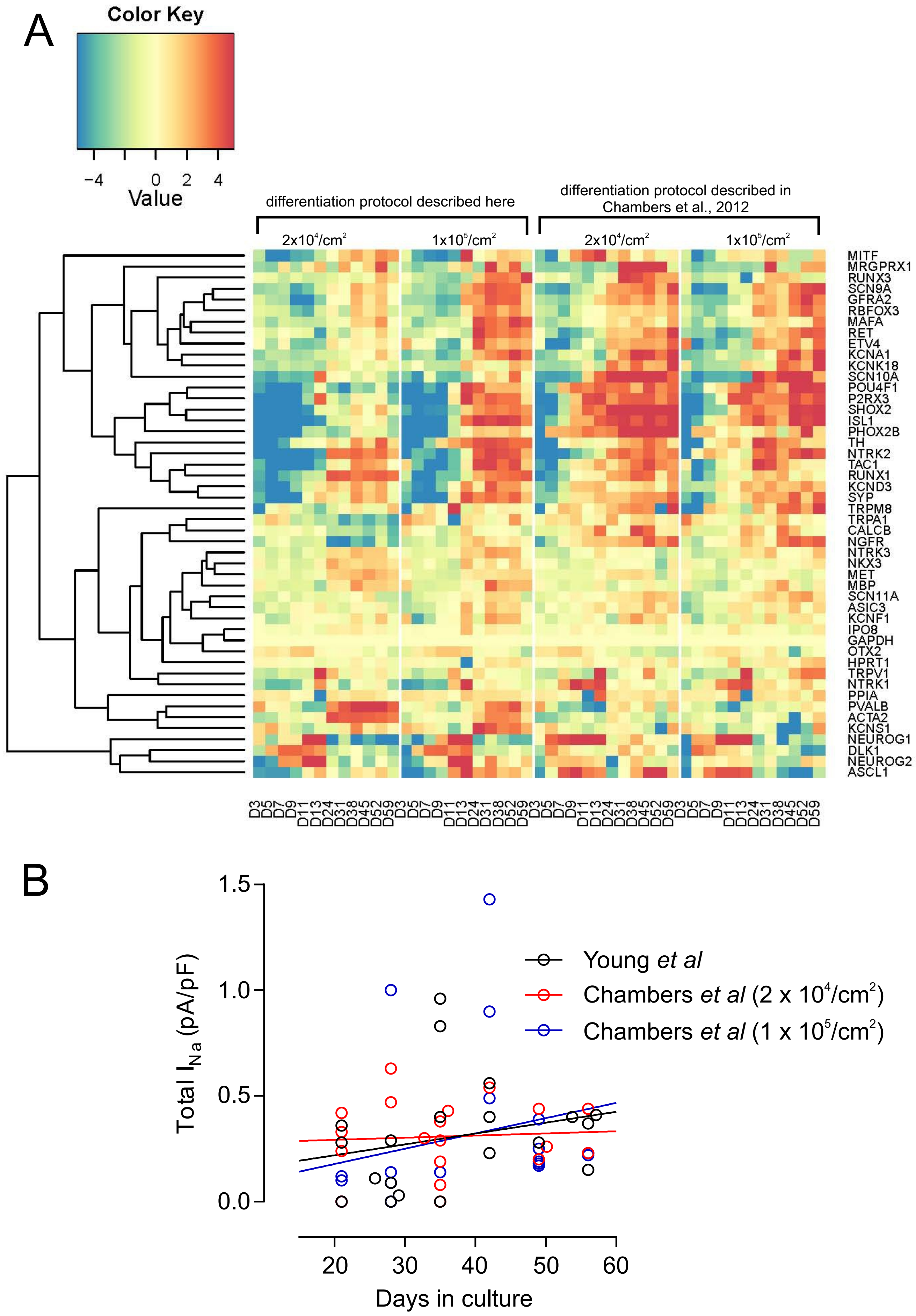
A
Color Key
−4
0
2
4
Value
differentiation protocol described here
differentiation protocol described in Chambers et al., 2012
2x10⁴/cm²
1x10⁵/cm²
2x10⁴/cm²
1x10⁵/cm²
MITF
MRGPRX1
RUNX3
SCN9A
GFRA2
RBFOX3
MAFA
RET
ETV4
KCNA1
KCNK18
SCN10A
POU4F1
P2RX3
SHOX2
ISL1
PHOX2B
TH
NTRK2
TAC1
RUNX1
KCND3
SYP
TRPM8
TRPA1
CALCB
NGFR
NTRK3
NKX3
MET
MBP
SCN11A
ASIC3
KCNF1
IPO8
GAPDH
OTX2
HPRT1
TRPV1
NTRK1
PPIA
PVALB
ACTA2
KCNS1
NEUROG1
DLK1
NEUROG2
ASCL1
D3 D5 D7 D9 D11 D13 D24 D31 D38 D45 D52 D59
B
Total I_Na (pA/pF)
0.0
0.5
1.0
1.5
20
30
40
50
60
Days in culture
Young et al
Chambers et al (2 x 10⁴/cm²)
Chambers et al (1 x 10⁵/cm²)
